# Photochemical activation of MH3-B1/rGel: a HER2-targeted treatment approach for ovarian cancer

**DOI:** 10.18632/oncotarget.3814

**Published:** 2015-04-14

**Authors:** Bente Bull-Hansen, Maria B. Berstad, Kristian Berg, Yu Cao, Ellen Skarpen, Ane Sofie Fremstedal, Michael G. Rosenblum, Qian Peng, Anette Weyergang

**Affiliations:** ^1^ Department of Radiation Biology, Institute for Cancer Research, Norwegian Radium Hospital, Oslo University Hospital, Oslo, Norway; ^2^ Immunopharmacology and Targeted Therapy Laboratory, Department of Experimental Therapeutics, M.D. Anderson Cancer Center, Houston, TX, USA; ^3^ Department of Biochemistry, Institute for Cancer Research, Norwegian Radium Hospital, Oslo University Hospital, Oslo, Norway; ^4^ Department of Pathology, Norwegian Radium Hospital, Oslo University Hospital, Oslo, Norway; ^5^ Current address: The Scripps Research Institute, Department of Chemistry, La Jolla, CA, USA

**Keywords:** ovarian cancer, HER2/neu/ErbB2, immunotoxin, photochemical internalization, photodynamic

## Abstract

HER2-targeted therapy has been shown to have limited efficacy in ovarian cancer despite frequent overexpression of this receptor. Photochemical internalization (PCI) is a modality for cytosolic drug delivery, currently undergoing clinical evaluation. In the present project we studied the application of PCI in combination with the HER2-targeted recombinant fusion toxin, MH3-B1/rGel, for the treatment of ovarian cancer. The SKOV-3 cell line, resistant to trastuzumab- and MH3-B1/rGel- monotherapy, was shown to respond strongly to PCI of MH3-B1/rGel to a similar extent as observed for the treatment-sensitive SK-BR-3 breast cancer cells. Extensive hydrolytic degradation of MH3-B1/rGel in acidic endocytic vesicles was indicated as the mechanism of MH3-B1/rGel resistance in SKOV-3 cells. This was shown by the positive Pearson's correlation coefficient between Alexa488-labeled MH3-B1/rGel and Lysotracker in SKOV-3 cells in contrast to the negative Pearson's correlation coefficient in SK-BR-3 cells. The application of PCI to induce the release of MH3-B1/rGel was also demonstrated to be effective on SKOV-3 xenografts. Application of PCI with MH3-B1/rGel was further found highly effective in the HER2 expressing HOC-7 and NuTu-19 ovarian cancer cell lines. The presented results warrant future development of PCI in combination with MH3-B1/rGel as a novel therapeutic approach in preclinical models of ovarian cancer.

## INTRODUCTION

Ovarian cancer is the most lethal of gynecologic cancers. It is often diagnosed at a late stage and the sensitivity to available anticancer therapeutics is highly limited. New treatment approaches are therefore warranted for the management of this disease. HER2 overexpression is most commonly associated with breast cancer where HER2 overexpression is found in 20-30 % of the cases [1]. Several HER2-targeted therapeutics have been approved for clinical use in the treatment of breast cancer. However, despite the success of HER2-targeted drugs in treating breast cancer, *de novo* as well as acquired resistance are major limitations in clinical practice [2], leaving patients with very limited treatment options. In case of ovarian cancer with known HER2 expression, several HER2-targeted drugs have been evaluated in clinical trials [3, 4, 5]. However, no HER2-targeted drug has so far been approved for clinical use, despite HER2 overexpression being reported in up to 35 % of all ovarian cancers [6, 7]. New HER2-targeted modalities with increased toxicity and less potential for development of resistance should therefore be an interesting approach for future treatment of ovarian cancer.

Increased toxicity of HER2-targeted drugs may be achieved through the utilization of single-chain HER2 antibody-based immunotoxins. Such constructs have been proven highly HER2 specific *in vitro* and induce considerable tumor growth delay in several animal models [8, 9, 10, 11]. The toxin component in such drugs acts by inhibition of protein synthesis and provides increased cytotoxic potential compared to clinically available HER2-targeted monoclonal antibodies (mAbs) and tyrosine kinase inhibitors (TKIs). Off-target cytotoxicity, which generally has been considered a major limitation for clinical use of immunotoxins, may be reduced by utilizing a type 1 ribosome-inactivating protein (RIP) [12]. In contrast to highly potent toxins such as ricin, Pseudomonas exotoxin (PE) and diphtheria toxin, type 1 RIPs lack a translocation domain which transports the toxin from endosomes into the cytosol [13]. Thus, a technology which allows improved endo/lysosomal release of these agents has the potential to increase specific cytotoxicity provided by type 1 RIP-based immunotoxins [14]. Photochemical internalization (PCI) is a technology which causes cytosolic release of drugs entrapped in endocytic vesicles [15, 16]. PCI is based on an amphiphilic photosensitizer (PS) which accumulates in the membranes of endosomes and lysosomes. Light exposure with appropriate wavelengths, excites the PS and initiates the production of reactive oxygen species (ROS) which in turn destroys the endo/lysosomal membrane [17]. PCI of several drugs has been proven as an effective treatment modality for cancer *in vivo* [18, 19, 20, 21] and ongoing clinical studies on PCI are showing highly promising results (www.clinicaltrials.gov; NCT01606566, NCT01872923, NCT01900158).

In the present study we evaluated PCI of the HER2-targeted single chain antibody-based recombinant immunotoxin MH3-B1/rGel in three ovarian cancer cell lines, generally resistant to HER2-targeted therapy, and also on ovarian cancer xenografts in athymic mice. These results indicate PCI of HER2-targeted toxins to be a promising treatment modality for HER2 overexpressing ovarian cancer and warrants future evaluation in preclinical models.

## RESULTS

### HER2 expression among the cell lines

**Figure 1:**
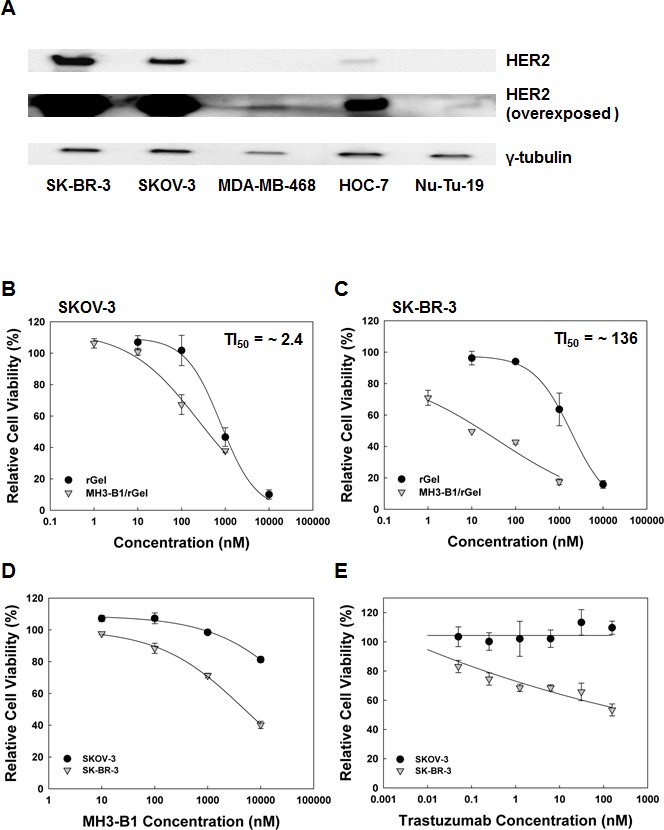
Cellular HER2 expression and cytotoxicity of HER2-targeted compounds (**A**). Western blot showing basal HER2 expression in SK-BR-3, SKOV-3, MDA-MB-468, HOC-7 and Nu-Tu-19 cells, representative blot of two experiments. Relative cell viability (MTT) of SKOV-3 and SK-BR-3 cells following 72 hrs incubation with MH3-B1/rGel and rGel (**B** and **C**), MH3-B1 (**D**) and trastuzumab (**E**), representative sigmoidal curves of three experiments (fit model: a/(1+exp(−(x-x_0_)/b))), error bars = SD.

The HER2 expression level in the 4 selected human cancer cell lines was found to vary in agreement with other reports. Both SK-BR-3 and SKOV-3 were found to be HER2-high expressing and the HER2 level in SK-BR-3 was indicated higher than observed in the SKOV-3 cells [10, 28] (Fig. 1A). An intermediate HER2 expression was found in the HOC-7 cell line [29] (Fig. 1A) while MDA-MB-468 was indicated as HER2-low [30, 10] (Fig. 1A). A weak HER2 band was also detected on overexposed western blots of Nu-Tu-19 cells (rat orgin) (Fig. 1A). However, the apparent weak HER2 expression in Nu-Tu-19 cells may be due to poor recognition of HER2 rat antigen by the antibody (antibody against human HER2) and comparison of HER2 level between Nu-Tu-19 and the human cell lines is therefore not possible.

### Sensitivity of SK-BR-3 and SKOV-3 cells to HER2-targeted therapeutics

In this study, the responsiveness to PCI of MH3-B1/rGel was primarily evaluated in 2 HER2 overexpressing cell lines; the human breast cancer cell line SK-BR-3 and the human ovarian cancer cell line SKOV-3 (Fig. 1A). Subjecting the 2 cell lines to a 72 hrs incubation of the HER2-targeted immunotoxin MH3-B1/rGel revealed a low TI_50_ of ~2.4 in the SKOV-3 cell line, compared to the TI_50_ of ~136 in SK-BR-3 cells (Fig. 1B and 1C). The lack of response to MH3-B1/rGel in SKOV-3 cells was also reflected in the response to the scFv targeting moiety MH3-B1 which reduced the viability by ~20 % in SKOV-3 cells compared to ~60 % in SK-BR-3 cells following a 72 hrs incubation at a 10 μM concentration (Fig. 1D). The SKOV-3 cells were in addition found resistant to the HER2-targeted mAb trastuzumab (Fig. 1E), in agreement with previous reports [29, 31] while the SK-BR-3 cells responded well with an LD_50_ of ~100nM. The present results therefore indicate that SKOV-3 cells are generally resistant to HER2-targeted antibody-based therapy.

### Uptake and cellular localization of Alexa488-MH3-B1/rGel in SK-BR-3 and SKOV-3 cells

**Figure 2:**
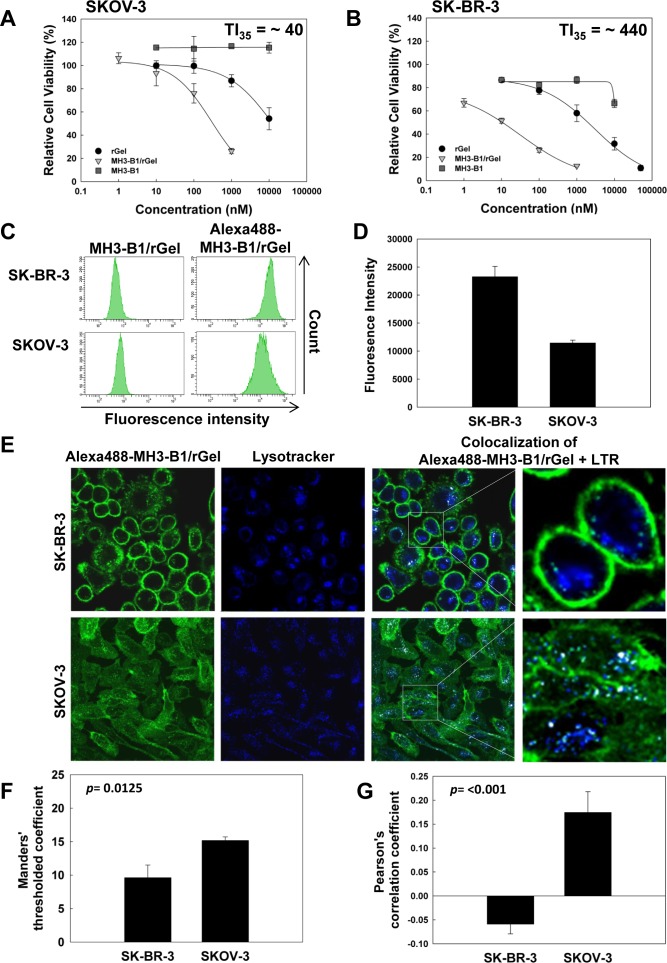
Cytotoxicity, uptake and cellular localization of MH3-B1/rGel after 4 hrs incubation Relative cell viability (MTT) of SK-BR-3 (**A**) and SKOV-3 (**B**) following 4 hrs incubation with MH3-B1/rGel, rGel and MH3-B1, representative sigmoidal curves of three experiments, (fit model: a/(1+exp(−(x-x_0_)/b))), error bars = SD. (**C**): Cellular uptake of 2 nM Alexa488-MH3-B1/rGel following 4 hrs incubation in SK-BR-3 and SKOV-3 cells, representative flow cytometry charts of three experiments. (**D**): Total cellular Alexa488-MH3-B1/rGel fluorescence, average of three experiments, error bars = SE. (**E**): SK-BR-3 and SKOV-3 cells following 4 hrs incubation of 2 nM Alexa-488-MH3-B1/rGel. Alexa488-MH3-B1/rGel (green), LTR (blue) and colocalization of the two (white). The confocal micrographs are representative of three independent experiments. The colocalization of Alexa488-MH3-B1/rGel with LTR was calculated by the Manders' thresholded coefficient from the total cellular accumulation measured (**F**) and the Pearson's correlation coefficient was used to calculate the correlation between Alexa488-MH3-B1/rGel and LTR based on fluorescence intensities/pixel (**G**), F and G shows average of three experiments, error bars = SE.

PCI is a method for cytosolic release of drugs entrapped in endosomes and lysosomes. PCI *in vitro* is usually performed by a 1-18 hrs incubation of the macromolecule of interest prior to light exposure [32, 17]. This relatively short incubation time of the macromolecule is utilized to avoid strong influence of lysosomal degradation on the PCI outcome. In the present *in vitro* protocol, a 4 hrs pulse of 2 nM MH3-B1/rGel was used prior to the light exposure. Since the intracellular trafficking of MH3-B1/rGel in the post-treatment period is expected to influence on the therapeutic effect, a cytotoxicity evaluation was performed 72 hrs after the 4 hrs pulse of MH3-B1/rGel to verify that SKOV-3 cells were resistant to MH3-B1/rGel also after a 4 hrs treatment (Fig. 2A and 2B). The TI's after 4 hrs treatment were found to be higher in both cell lines compared to that after 72 hrs treatment. However, the SKOV-3 cells were found to be relatively less sensitive to the treatment as e.g. 2 nM MH3-B1/rGel reduced cell viability by ~35 % in SK-BR-3 cells (Fig. 2B), but did not induce detectable cytotoxicity in the SKOV-3 cell line (Fig. 2A). Calculating the TI at IC_35_ of MH3-B1/rGel vs. rGel (TI_35_) revealed an ~11-fold higher TI_35_ in SK-BR-3 cells compared to SKOV-3 cells after 4 hrs treatment with MH3-B1/rGel. SKOV-3 cells were therefore found resistant to MH3-B1/rGel also when administrated for 4 hrs compared to SK-BR-3 cells. Four hrs incubation of MH3-B1 (without rGel) did not induce any significant cytotoxicity in any of the cell lines, indicating MH3-B1/rGel-mediated toxicity to be generated through ribosomal inhibition (Fig. 2A and 2B).

MH3-B1/rGel primarily exerts its intracellular action by inducing inhibition of protein synthesis. The mechanism of MH3-B1/rGel resistance in the SKOV-3 cell line could be due to differences in overall uptake and/or intracellular MH3-B1/rGel localization and trafficking compared to SK-BR-3 cells. Subjecting SK-BR-3 and SKOV-3 cells to a 4 hrs incubation of 2 nM Alexa488-labeled MH3-B1/rGel revealed a ~2-fold higher Alexa488-MH3-B1/rGel uptake in SK-BR-3 cells compared to SKOV-3 cells (Fig. 2C and 2D). However, this 2-fold difference could only partly explain the ~11-fold higher TI_35_ in SK-BR-3 cells following a 4 hrs MH3-B1/rGel incubation (Fig. 2A and 2B).

The MH3-B1/rGel construct must eventually translocate from endosomes and/or lysosomes into the cytosol to exert its action on the ribosomes and avoid lysosomal degradation [12]. Thus, differences in distribution of the targeting toxin in endocytic vesicles between SKOV-3 and SK-BR-3 cells could explain the difference in MH3-B1/rGel sensitivity. Intracellular localization of Alexa488-labeled MH3-B1/rGel was detected by confocal microscopy which confirmed both binding of the immunotoxin to the plasma membrane and cellular internalization to endocytic compartments in both cell lines (Fig. 2E). The association of Alexa488-MH3-B1/rGel to the plasma membrane appeared much stronger in the SK-BR-3 cells than in the SKOV-3 cells. However, this visual appearance may at least partly be due to the more rounded up morphology of the SK-BR-3 cells. Diffuse fluorescence was, in addition, observed in the cytoplasm of SKOV-3 cells. The thin morphology of SKOV-3 cells (~1.5 μm), together with the axial resolution of the microscope (0.56 μm) and differences in the optimal plane of microscopy between cells in the same micrograph was, however, found as the reason for this diffuse fluorescence which was concluded as noise from the plasma membrane. A significantly higher fraction (15.2 % vs. 9.6 %) of Alexa488-MH3-B1/rGel was found to colocalize with LTR in SKOV-3 cells than in SK-BR-3 cells (*p* = 0.0125) (Fig. 2F). The distribution of Alexa488-MH3-B1/rGel correlated also better with the distribution of LTR as revealed by the Pearson's correlation coefficient of 0.17 (±0.04) in SKOV-3 cells as compared to −0.059 (±0.02) in the SK-BR-3 cells (*p* < 0.001) (Fig. 2G). A higher distribution of MH3-B1/rGel in acidic endocytic vesicles in SKOV-3 cells indicates a higher hydrolytic degradation of the immunotoxin in the vesicles of these cells compared to SK-BR-3 cells. This hydrolysis may take place in all acidic endocytic vesicles including both endosomes and lysosomes [33, 34]. Attempts have been made to remove plasma membrane associated fusion toxin in order to quantitatively measure intracellular fusion toxin. Such a method would make it possible to compare the impact of e.g. a lysosomotropic agent on MH3-B1/rGel degradation in the two cell lines. However, the high plasma membrane association of MH3-B1/rGel in these two cell lines makes this a difficult task since only small contamination from the plasma membrane is likely to influence strongly on this suggested experiment. Thus, the increased distribution of Alexa488-MH3-B1/rGel in acidic vesicles in the SKOV-3 cell line together with the higher overall uptake in the SK-BR-3 cells may explain the lower sensitivity to MH3-B1/rGel in the SKOV-3 cells compared to SK-BR-3 cells.

### PCI of MH3-B1/rGel in SK-BR-3 and SKOV-3 cells

**Figure 3:**
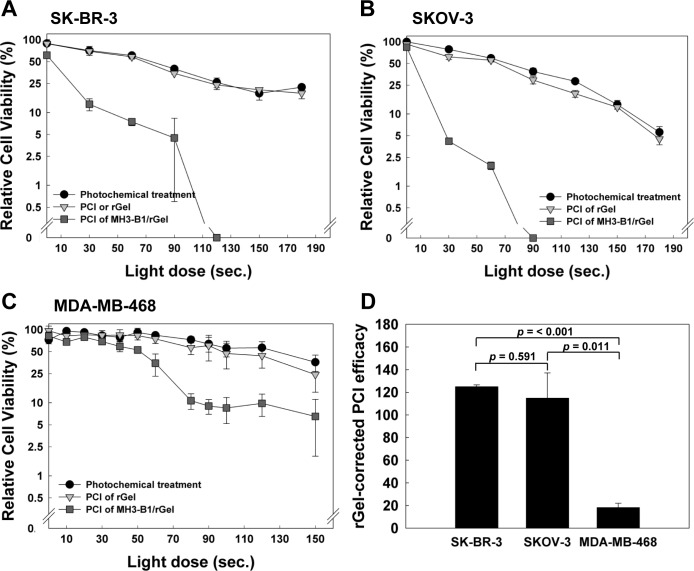
PCI efficacy of MH3-B1/rGel in SK-BR-3 and SKOV-3 cells Relative cell viability (MTT) of SK-BR-3 (**A**), SKOV-3 (**B**) and MDA-MB-468 (**C**) cells after PCI of 2 nM MH3-B1/rGel (incubated for 4 hrs), representative curves from three experiments, error bars = SD. (**D**): rGel-corrected PCI efficacy, average of three experiments, in SK-BR-3, SKOV-3 and MDA-MB-468 cells.

Since high hydrolytic degradation was indicated as a potential mechanistic cause of the low MH3-B1/rGel sensitivity in the SKOV-3 cells, we hypothesized that PCI may be useful in addressing this mechanism of resistance. Studies combining PCI and MH3-B1/rGel demonstrated highly effective cytotoxicity against SKOV-3 cells and appeared to reduce the cell viability to the same extent as observed for the SK-BR-3 cells (Fig. 3A and 3B). PCI of MH3-B1/rGel was also indicated as HER2 selective when compared to PCI of non-targeted rGel (Fig. 3A and 3B) and also when comparing the PCI data for SK-BR-3 and SKOV-3 cells with those obtained with the HER2 low expressing cell line MDA-MB-468 (Fig. 3A-3C). The efficacy of PCI of MH3-B1/rGel has recently been shown to correlate well with HER2 expression [23]. PCI efficacy may be established by comparing the light dose needed to obtain LD_50_ with PCI with that of the photochemical treatment (PS and light) and correct the calculation for cellular differences in sensitivity to the non-targeted toxin (as described in Materials and Methods, [23]). The PCI efficacy corrected for rGel-sensitivity in the two cell lines revealed similar efficacy of PCI of MH3-B1/rGel in the resistant SKOV-3 cells as in the SK-BR-3 cells (p > 0.05) (Fig. 3D). The rGel-corrected PCI efficacy in MDA-MB-468 cells was, however, found to be 6.8- and 6.3-fold reduced compared to that in SK-BR-3 and SKOV-3 cells respectively (Fig. 3D).

### PCI of MH3-B1/rGel in HOC-7 and NuTu-19 cells

**Figure 4:**
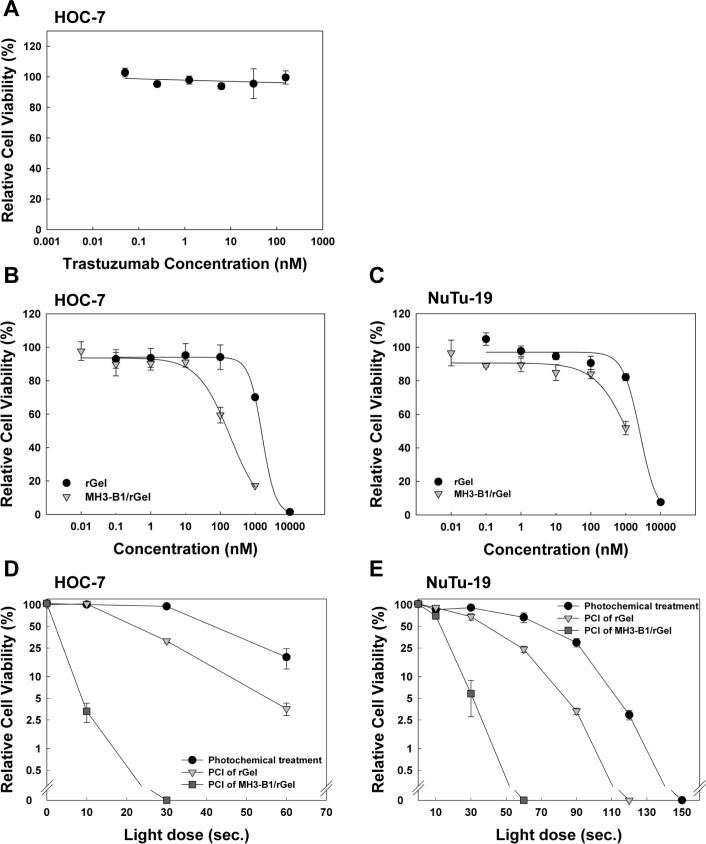
PCI of MH3-B1/rGel in HOC-7 and NuTu-19 cells Relative cell viability (MTT) of HOC-7 cells following 72 hrs incubation with trastuzumab (**A**) and MH3-B1/rGel and rGel (**B**). Relative cell viability (MTT) of NuTu-19 cells following 72 hrs incubation with MH3-B1/rGel and rGel (**C**). Representative sigmoidal curves of three experiments (fit model: a/(1+exp(−(x-x_0_)/b)), error bars = SD. (**D**) and (**E**) shows relative cell viability (MTT) of HOC-7 and NuTu-19 cells after PCI of 2nM MH3-B1/rGel (incubated for 4 hrs), representative curves of three experiments, error bars = SD.

Ovarian cancer is generally known to have low sensitivity to HER2-targeted therapeutics despite strong HER2 expression [3, 5]. The high PCI-induced efficacy of MH3-B1/rGel in SKOV-3 cells was therefore also tested in the ovarian cancer cell lines HOC-7 and NuTu-19. HOC-7 cells were found resistant to trastuzumab and MH3-B1/rGel to a comparable level as detected in SKOV-3 cells (Fig. 4A and 4B, Fig. 1B and 1E). Accordingly, NuTu-19 cells have previously been reported to not respond to trastuzumab [35] and were also found resistant to MH3-B1/rGel (Fig. 4C). PCI was shown to increase the effect of MH3-B1/rGel significantly in HOC-7 and Nu-Tu-19 cells (Fig. 4D and 4E), indicating that MH3-B1/rGel is accumulating specifically in both cell lines although the therapeutic specificity is more strongly seen after activation by PCI. These results are in accordance with those seen in the SKOV-3 cells (Fig. 3B). HER2 expression in HOC-7 cells was, however, much lower than observed for SKOV-3 cells (Fig. 1A).

### PCI of MH3-B1/rGel on SKOV-3 xenografts *in vivo*

**Figure 5:**
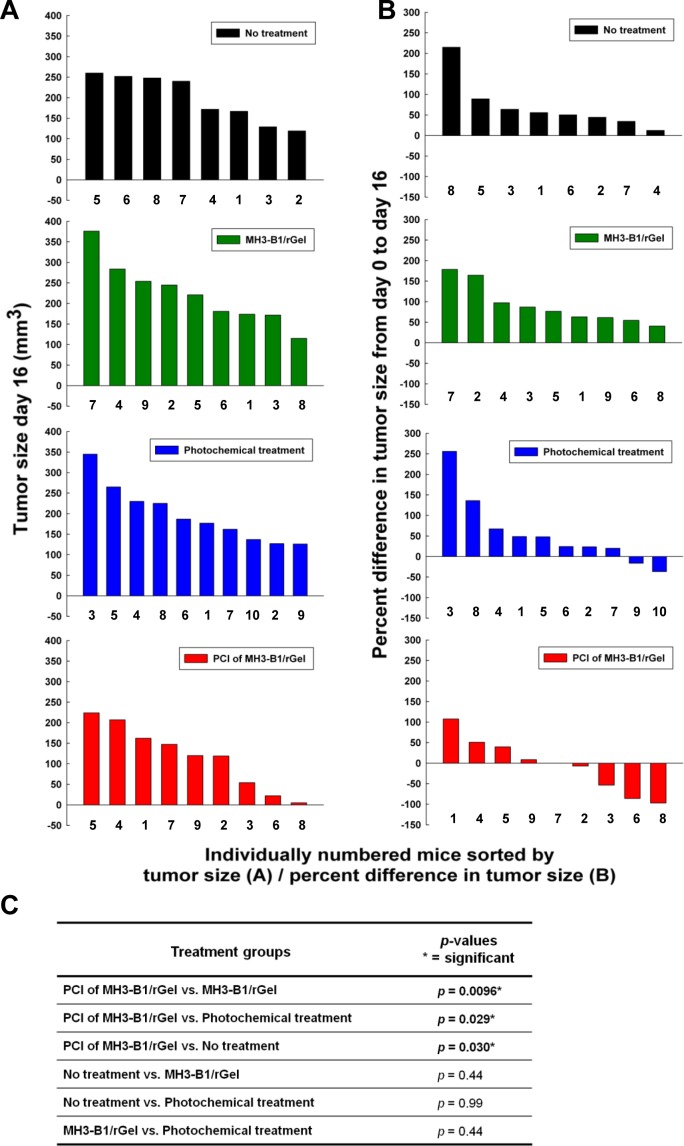
Tumor size after PCI of MH3-B1/rGel in SKOV-3 xenografts The bars show the measured tumor size at day 16 for each animal in the four treatment groups (**A**) and the percent change in tumor size at day 16 calculated from the measured tumor size at treatment day (day 0) for each animal in the four treatment groups (**B**). Calculated *p*-values representing statistical differences in tumor size between the treatment groups at day 16 post treatment (**C**).

**Figure 6:**
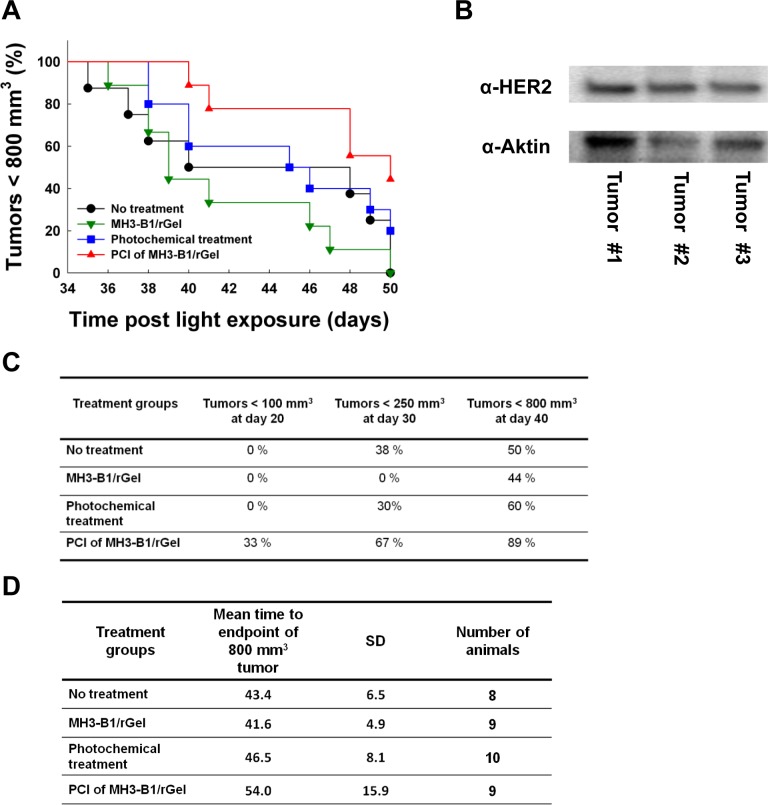
Treatment response following PCI of MH3-B1/rGel in SKOV-3 xenografts Kaplan Meyer plot illustrating response from day 34-50 in the four treatment groups (**A**). Tumor volume end point = 800 mm^3^. Western blot of HER2 in three SKOV-3 tumors (**B**). Percent of animals bearing tumors < 100 mm^3^ at day 20, < 250 mm^3^ at day 30 and < 800 mm^3^ at day 40 post light exposure in the four treatment groups (**C**). Average time to reach the endpoint of 800 mm^3^ tumor volume in the four treatment groups (**D**).

The high efficacy of PCI of MH3-B1/rGel in the MH3-B1/rGel resistant SKOV-3 cells was further evaluated on SKOV-3 subcutaneous xenografts in athymic mice. This is a relatively slow growing tumor model and the mean time to reach the endpoint of 800 mm^3^ from a treatment volume of ~100 mm^3^ was 43.4 days (Fig. 6D). The photochemical treatment induced edema and accurate measurements of tumors were therefore difficult to obtain the first ~2 weeks after treatment. Early effects on tumor growth following the different treatments were assessed on day 16 since this was the first day without any detectable edema in any of the animals. Comparing the size of all tumors at day 16 revealed significantly smaller tumors in the PCI group compared to the control groups receiving either MH3-B1/rGel, photochemical treatment or no treatment (Fig. 5A and 5C). No significant difference was found between the no treatment group and the groups receiving MH3-B1/rGel or photochemical treatment as monotherapies. Neither was there any difference in tumor size between the group receiving MH3-B1/rGel and the photochemical treatment group at day 16 (Fig. 5A and 5C).

The percent change in tumor size at day 16 compared to the tumor size measured at day 0 for each animal, revealed a stronger inhibition of tumor growth in the PCI group than in the control groups. All the animals in the MH3-B1/rGel monotherapy and no treatment group showed an increase in tumor volume at day 16 compared to day 0 (Fig. 5B). However, the tumors subjected to the photochemical treatment (PS and light) showed an overall lower growth and even some reduction in tumor volume. The volume of the tumors in the PCI group was even smaller than those only subjected to the photochemical treatment (PS and light) and a higher number of tumors showed a reduced tumor volume.

Although statistically not significant, a treatment response of SKOV-3 tumors following PCI of MH3-B1/rGel was also indicated in Kaplan-Meier plots between day 38 and 50 post light exposure (Fig. 6A). At day 50, 44 % of the animals that received PCI of MH3-B1/rGel had tumors with a size < 800 mm^3^ compared to 20 % in the photochemical treatment group, whereas all the tumors in the no treatment group and the group that received MH3-B1/rGel monotherapy had reached the end point of 800 mm^3^ (Fig. 6A). Measurements of tumor growth delay confirmed treatment response in the PCI group compared to the control groups also at day 20, 30 and 40 post-treatment as illustrated in Fig. 6C. The mean time to reach a tumor volume of 800 mm^3^ is presented in Fig. 6D and growth curves is presented in S1. No correlation was found between the actual tumor size at the day of treatment and treatment response.

The effect of the applied PCI treatment was smaller than expected from the encouraging *in vitro* data presented here in addition to previous *in vivo* reports on PCI of other targeting toxins [20, 21]. The 48 hrs interval between MH3-B1/rGel administration and light exposure used in this study was based on MH3-B1/rGel accumulation studies in BT474 M1 tumors [9] and this interval was decreased to 24 hrs to study if this would increase the treatment response. No increase in efficacy was, however, detected by reducing the MH3-B1/rGel-to-light interval to 24 hrs (data not shown). Western blots of HER2 in three SKOV-3 tumors verified high expression of HER2 in these xenografts (Fig. 6B).

### Tumor accumulation of MH3-B1/rGel in SKOV-3 xenografts

The amount of MH3-B1/rGel localized to the tumor at the time of light exposure (48 hrs post injection) was assessed by western blotting of tumor lysates. These western analysis failed, however, to demonstrate the presence of the fusion construct in the tumors (results not shown). A standard of MH3-B1/rGel indicated 0.5 ng MH3-B1/rGel as the detection limit for MH3-B1/rGel on a western blot which would correspond to 208 ng (3.78 x10^−12^ mol) MH3-B1/rGel/g tumor tissue in a 0.2 g tumor. The amount of MH3-B1/rGel localized to the tumors at the time of light exposure with the present protocol was therefore less than 3.78 × 10^−12^ moles/g tumor tissue.

### Adverse effects of the treatment

**Figure 7:**
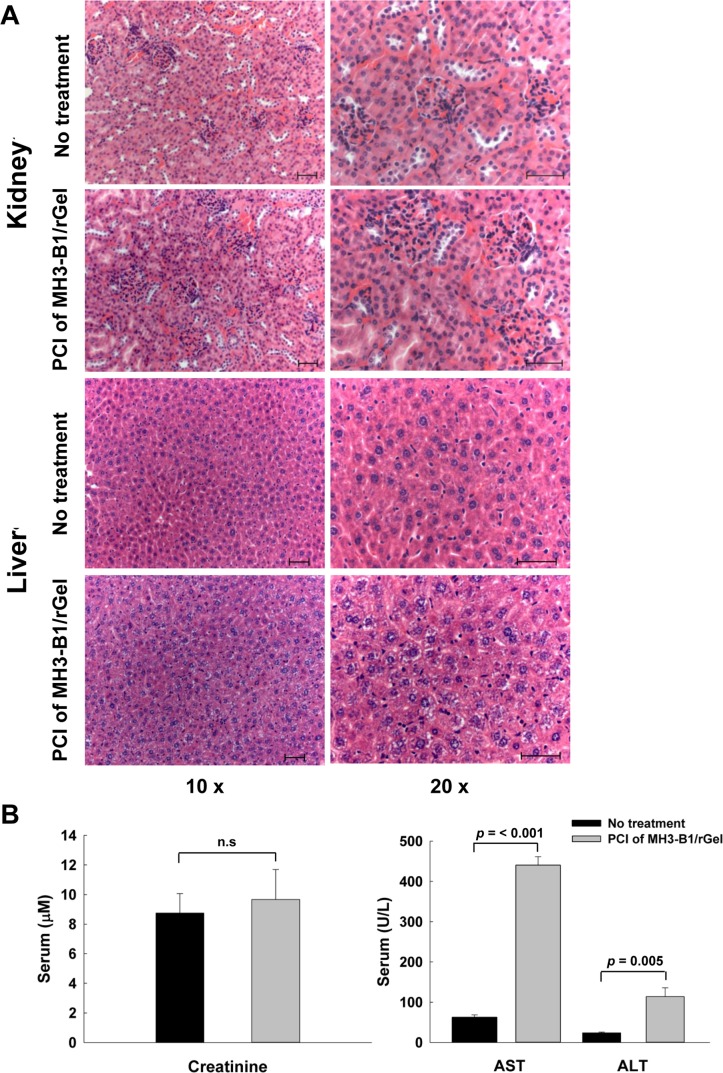
Adverse effects following PCI of MH3-B1/rGel in SKOV-3 xenografts H&E staining of kidney and liver tissue in untreated and PCI of MH3-B1/rGel treated animals 24 hrs post light exposure (**A**). Bar: 50 μm. Serum levels of creatinine, AST and ALT in untreated and PCI of MH3-B1/rGel treated animals 24 hrs post light exposure (**B**).

Damage to the liver and kidneys of mice was evaluated 24 hrs after the PCI treatment. No adverse effects on the kidneys were noted by histopathology and also by measuring the serum levels of creatinine, an indicator of renal function (Fig. 7A and 7B). Hepatocytes, however, showed their central nuclei surrounded by many vacuoles in the cytoplasm (Fig. 7A), a typical morphological alteration indicating reversible degeneration. This is consistent with the results which demonstrated a significant increase in serum levels of AST and ALT (Fig. 7B) in the PCI-treated animals. Such reversible hepatic degeneration indicates that the PCI treatment may induce a slight liver toxicity, but with no reduction in the daily activities as well as body weight of the treated mice. In the skin, erythema for up to 2 weeks was detected at the illuminated area, while light alone (without PS) as a control did not cause such effect. In addition, a small wound in the treated area was observed in 3 out of 10 mice, but leaving no scarring. Thus, generally, PCI of MH3-B1/rGel was considered as a tolerable treatment.

## DISCUSSION

In the present study, we have utilized the intracellular drug delivery technology PCI to potentiate the cytotoxicity of the HER2-targeted recombinant immunotoxin, MH3-B1/rGel in HER2 overexpressing ovarian cancer cell lines. Ovarian cancer is the most lethal of all gynecological cancers with a 5 year survival of only 44.2% [36]. Up to 35% of ovarian cancers have been reported as HER2-positive [37, 6, 7]. HER2-targeted antibodies and molecular inhibitors have, however, failed to demonstrate significant clinical benefit as monotherapy [38] and the prognostic value of HER2 expression in ovarian cancer is controversial [38]. The aim of this study was to utilize HER2 as the delivery portal for MH3-B1/rGel to endocytic vesicles in the ovarian cancer cell line SKOV-3. PCI-induced cytosolic translocation of MH3-B1/rGel should further facilitate ribosomal binding, inhibition of protein synthesis and induction of cell death. The HER2-positive breast cancer cell line SK-BR-3, recently reported as highly sensitive to PCI of MH3-B1/rGel [23] was used as a positive control throughout the study.

Both SKOV-3 and SK-BR-3 cells were found to be HER2-positive, although HER2 was more pronounced in SK-BR-3 cells in agreement with a previous report [39]. The increased level of HER2 found in SK-BR-3 cells could, however, not explain the ~60-fold higher TI (72 hrs incubation) of MH3-B1/rGel compared to the SKOV-3 cells or the significantly decreased sensitivity to MH3-B1 or trastuzumab in SKOV-3 cells. The mechanism of MH3-B1/rGel toxicity is dual because it (i): inhibits HER2 signaling and function through MH3-B1 and (ii): induces toxicity through the rGel payload which acts intracellulary by inactivating ribosomes leading to a generalized inhibition of protein synthesis. SKOV-3 cells have also previously been reported resistant to trastuzumab [28, 31]. Trastuzumab resistance in SKOV-3 cells is shown to depend on a constitutive activation of AKT, caused by lack of the tumor suppressor protein RAS homolog member I and also on low expression of PTEN which is recruited to the plasma membrane upon trastuzumab–HER2 association and inhibits AKT as one of the main mechanisms of trastuzumab induced G1 arrest [28, 40]. MH3-B1 binds to another domain of HER2 than trastuzumab (results not published). The present results show, however, that SKOV-3 cells are resistant also to MH3-B1. AKT inhibition post MH3-B1 treatment was not assessed in the present study, and has not been reported before. However, the observed cross-resistance between trastuzumab and MH3-B1 may indicate a partial overlapping mechanism of resistance between these two HER2-targeted compounds.

In our study, 72 hrs treatment of MH3-B1/rGel in SKOV-3 cells induced only minor toxicity by HER2 inhibition as shown by the MH3-B1-induced reduction in viability at comparable concentrations. Four hrs incubation of MH3-B1 did not induce any reduction in viability in SKOV-3 or SK-BR-3 cells at doses < 10 μM (Fig. 2A and 2B). The ~11-fold increased TI of MH3-B1/rGel in SK-BR-3 cells compared to the SKOV-3 cell line after a 4 hrs incubation of the toxins therefore indicate that SKOV-3 cells are resistant also to the gelonin-induced toxicity of MH3-B1/rGel.

PCI is a method for cytosolic release of therapeutics entrapped in endocytic vesicles [17]. Endocytic trapping and subsequent hydrolytic degradation of MH3-B1/rGel as an important mechanism of resistance in SKOV-3 cells was shown by the efficacy of PCI in this cell line. The similar efficacy of PCI of MH3-B1/rGel (rGel-corrected PCI efficacy) in SKOV-3 and SK-BR-3 cells found (Fig. 3D), despite the ~2-fold higher MH3-B1/rGel accumulation in SK-BR-3 cells (Fig. 2D), also suggests a higher distribution of MH3-B1/rGel to endocytic vesicles in SKOV-3 cells. The higher distribution of MH3-B1/rGel in acidic vesicles in SKOV-3 cells is also reflected in the higher Pearson's correlation coefficient of MH3-B1/rGel and LTR found in this cell line (Fig. 2G). A more detailed mechanistic explanation on MH3-B1/rGel resistance should be addressed in future studies. These studies may include siRNA targeting different cathepsins and also inhibitors of hydrolytic activity.

PCI of MH3-B1/rGel on SKOV-3 xenografts in nude mice also revealed significantly smaller tumors in the PCI group compared to all control groups when measured 16 days after light exposure. This *in vivo* effect was smaller than expected based on the presented *in vitro* results and also on other reports on PCI of gelonin-based immunotoxins [20, 21]. It has previously been shown that as little as 6.3 × 10^−12^ moles/g tumor tissue is sufficient for complete remission with an immunotoxin based on PE (anti-Tac(Fv)-PE38) [41]. We have tried, by western blotting, to evaluate the amount of MH3-B1/rGel present in SKOV-3 tumors 48 hrs post i.v. injection of doses of 2 mg/kg (0.05 mg/mouse). We believe that this dose was likely below the limit of detection of our western analysis which was found to correspond to 3.78 x10^−12^ moles/g tumor tissue. Compared to PE, rGel lacks a domain for cytosolic translocation [13]. If PCI can induce a 100% cytosolic release of MH3-B1/rGel, the 2 mg/kg dose administered 48 hrs prior to light exposure is still significant less than what has previously been indicated as a curative dose of PE. However, we find it highly promising that the small amount of tumor-localized MH3-B1/rGel appears to demonstrate antitumor activity in combination with PCI.

In the present study, only a single i.v. injection at a dose of 2 mg/kg was used. The increased efficacy provided by PCI has previously been shown to induce complete responses following only one injection of gelonin-based immunotoxins at comparable doses as here applied, however, the lack of severe adverse effects following 2 mg/kg of MH3-B1/rGel suggests that this dose may be increased to improve the PCI-induced efficacy. A significant effect on tumor growth delay has previously been reported after a fractionated dose (6 injections in 10 days) of 24 mg/kg MH3-B1/rGel [9]. This report therefore warrants a demonstration of higher single doses of MH3-B1/rGel, than currently available, in combination with PCI.

Altogether, the presented data suggest PCI of MH3-B1/rGel as a HER2-targeted treatment approach for HER2-positive cancers, including ovarian cancers resistant to HER2-targeted therapeutics. We have recently reported that PCI of MH3-B1/rGel exerts HER2-induced toxicity also in breast cancer with low HER2 expression [23]. PCI of MH3-B1/rGel may therefore be beneficial not only to the ~35% of ovarian cancers with high HER2 expression, but also in cases with medium to low HER2 expression. Although also effective in HER2-low expressing cancer, the PCI-induced efficacy of MH3-B1/rGel clearly correlates with HER2 expression [23] (Fig. 3). Off-target effects of the treatment in HER2 positive normal cells should, in addition, be of minor importance since off-target tissue in general (except in the liver) will accumulate less PS [42] and will not be subjected to light exposure. The present report warrants further evaluation in preclinical ovarian cancer models. These studies will also include pharmacologic evaluation to conclude on the mechanisms of cell death induced by the treatment. The *in vivo* protocol must, however, be optimized with respect to MH3-B1/rGel dose.

## MATERIALS AND METHODS

### Cell lines and cultivation

Four HER2 overexpressing cell lines were used in this study. The ovarian adenocarcinoma cell line SKOV-3 (ATCC, HTB-77™), purchased from American Type Culture Collection (ATCC, Manassas, VA, USA), the human breast adenocarcinoma cell line SK-BR-3, kindly provided by the Department of Biochemistry, Institute for Cancer Research, Norwegian Radium Hospital, Oslo University Hospital, Oslo, Norway, the human ovarian carcinoma cell line HOC-7, kindly provided by Dr. Yvonne Anderson, Department of Tumor Biology, Institute for Cancer Research, Norwegian Radium Hospital, Oslo University Hospital, Oslo, Norway, and the rat ovarian cancer cell line NuTu-19, originally a gift from Dr. A.L Major, University of Geneva, Switzerland [22]. The HER2 low expressing human breast adenocarcinoma cell line MDA-MB-468 (ATCC, HTB-132^TM^) was obtained from ATCC. SKOV-3 and SK-BR-3 cells were cultured in McCoy's 5A medium while HOC-7 and NuTu-19 cells were cultured in RPMI 1640 medium. Both media were obtained from Sigma-Aldrich (St. Louis MO) and modified as previously described [23]. The MDA-MB-468 cells were cultured in Leibovitz`s L-15 medium (Lonza, Basel, Switzerland) modified as previously described [23] with free gas exchange with atmospheric air.

### Preparation of MH3-B1 and MH3-B1/rGel

The HER2-targeted single chain antibody-fragment MH3-B1 and the HER2-targeted recombinant fusion toxin MH3-B1/rGel were produced as previously reported [9]. rGel is the recombinant version of the type I RIP gelonin.

### Evaluation of cytotoxicity

The MTT viability assay was in the present study used to assess cytotoxicity of the different treatments. Cytotoxicity data, as measured by clonal cell survival, has previously been compared with MTT data as obtained here [24, 25]. No significant difference has been observed in these studies.

### Evaluation of HER2-targeted therapeutics

Cells were seeded at 2.5×10^3^ (SKOV-3), 6.0×10^3^ (SK-BR-3), 2×10^3^ (HOC-7) and 1.5×10^3^ (NuTu-19) cells/well in 96-well plates (Nunc) and allowed to attach to the substratum overnight. The cells were then subjected to a 72 hrs incubation of increasing concentrations of trastuzumab (Hoffmann-La Roche, Basel, Switzerland) or the scFv MH3-B1. To examine the cytotoxicity of the immunotoxin MH3-B1/rGel, the cells were incubated with MH3-B1/rGel or rGel for 4 or 72 hrs and the MTT viability assay was done 72 hrs post treatment initiation for both incubation procedures as previously reported [23]. IC_50_ (72 hrs incubation) or IC_35_ (4 hrs incubation) values of MH3-B1/rGel and rGel were calculated from sigmoidal curves (see 2.11) and used for establishing the targeting index (TI) of MH3-B1/rGel:

$$T\arg eting\;index_{IC50}\;=\;\frac{IC_{50}rGel}{IC_{50}MH3-B1/rGel}$$

*Formula for calculating the TI at IC_50_ (TI_50_) for rGel and MH3-B1/rGel. The TI at IC_35_ (TI_35_) was assessed by the same formula inserting the IC_35_ values.

### Light source

The cells were exposed to light from LumiSource^®^ (PCI Biotech AS, Oslo, Norway), delivering blue light (λ_max_ = 435 nm) from a bank of four 18W Osram L 18/67 light tubes with an irradiance of 12.6 mW/cm^2^, varying less than 10 % across the illumination area.

### PCI of MH3-B1/rGel *in vitro*

The PS TPCS_2a_ (meso-tetraphenyl chlorin with two sulfonate groups on adjacent phenyl rings, Amphinex^®^) was provided by PCI Biotech AS. Experiments with TPCS_2a_ were performed under subdued light. Cells were seeded at 10×10^3^ (SK-BR-3), 5×10^3^ (SKOV-3), 3×10^3^ (HOC-7) and 2×10^3^ (NuTu-19) cells/well in 96-well plates (Nunc) and allowed to attach for 6 hrs. MDA-MB-468 were seeded at 8×10^3^ cells/well and attached over night. Cells were then subjected to 0.4 μg/ml (SK-BR-3, SKOV-3, HOC-7 and NuTu-19) or 0.1 μg/ml (MDA-MB-468) TPCS_2a_ for 18 hrs, washed twice with culture medium and incubated with 2 nM MH3-B1/rGel for 4 hrs before the medium was replaced with new drug-free culture medium and the cells exposed to increasing doses of light from LumiSource^®^. All treatment regimens were performed in triplicates and the MTT assay was used for cell viability evaluation 48 hrs (SK-BR-3, HOC-7, NuTu-19 and MDA-MB-468) or 96 hrs (SKOV-3) after light exposure as previously reported [23]. PCI efficacy was corrected for cellular sensitivity to rGel treatment as follows [23]:

$$rGel\;-\;corrected\;PCI\;efficacy(LD_{50})\;=\;\frac{PCI\;efficacy\;\times \;IC_{50}rGel}{100}$$

where PCI efficacy is described as:

$$PCI\;efficacy\;=\;\frac{photochemical\;treatment\;LD_{50}}{PCI\;LD_{50}}$$

where the photochemical treatment is the combination of PS and light without any protein toxin added and IC_50_ rGel refers to the rGel concentration in nM.

Thus,

$$rGel\;-\;corrected\;PCI\;efficacy(LD_{50})\;=\;\frac{photochemical\;treatment\;LD_{50}\;\times \;IC_{50}rGel}{PCI\;LD_{50}\;\times \;100}$$

### Western blot analysis

For assessment of HER2 expression, sample preparation and western blotting were performed as previously described [23]. Three different substrate detection systems were used on the Western blots dependent on the band signal; LumiGlo chemiluminescent substrate system (KPL, Gaithesburg, MD USA), Supersignal West Dura Extended duration Substrate (Thermo Scientific, Rockford, IL, USA) and Supersignal West Femto Maximum Sensitivity Substrate (Thermo Scientific). Unfortunately, we were not able to find any antibody recognizing rat HER2.

For detection of HER2 and MH3-B1/rGel in SKOV-3 tumors, MH3-B1/rGel was injected i.v. at 2 mg/kg when the tumors reached 200mm^3^. The tumors were harvested 48 hrs post injection and prepared as previously described [26]. Prior to SDS-PAGE, the samples were mixed with 5x reducing buffer and western blotting was performed as recently described [23]. The primary anti-gelonin polyclonal antibody was established by the Rosenblum lab.

### Cellular uptake and localization of Alexa488-MH3-B1/rGel

Alexa488-labeled MH3-B1/rGel was prepared as recently described [23]. For evaluation of cellular uptake of Alexa488-MH3-B1/rGel, cells were seeded at 4×10^5^ (SK-BR-3) and 3×10^5^ (SKOV-3) cells/well in 6-well plates and treated and analyzed as previously reported by a BD FACSCalibur™ flow cytometer and BD CellQuest™ software (Becton-Dickinson) [23].

Alexa488-MH3-B1/rGel was also used to evaluate the cellular localization of MH3-B1/rGel by confocal microscopy in the presence of Lysotracker^®^ (LTR) as recently reported [23].

To calculate the degree of overlap between the chromophores Alexa488-MH3-B1/rGel and LTR, the weighted colocalization coefficient Manders' thresholded coefficient (MTC) was used. The calculation of the MTC designates the sum of intensities of colocalizing pixels in the respective channel, as compared to the overall sum of pixel intensities above threshold and formulated by:

$$MTC\;=\;\frac{\sum ch1_{i,coloc}}{\sum ch1_{i}}$$

where Ch1*_i,coloc_* is the fluorescence intensity of Alexa488-MH3-B1/rGel in each pixel where the intensity of LTR is above threshold, and ΣCh1_i_ is the sum of Alexa488-MH3-B1/rGel pixel intensities above threshold in the cells. A threshold was set above the noise level for each chromophore, and was determined by imaging control cells in the absence of LTR and PS. The same threshold was used in all cells [27].

The Pearson's correlation coefficient (PCC) was used to calculate the correlation between the fluorescence intensity of Alexa488-MH3-B1/rGel and LTR where a value of 1 represents 100 % positive correlation, 0 indicates no correlation and −1 represents a total negative correlation formulated by:

$$=\;\frac{\sum \left(ch1_{i}\;-\;ch1_{avg}\right)\left(ch2_{i}\;-\;ch2_{avg}\right)}{\sqrt{\sum \left({\left(ch1_{i}\;-\;ch1_{avg}\right)}^{2}{\left(ch2_{i}\;-\;ch2_{avg}\right)}^{2}\right)}}$$

where Ch1_i_ and Ch2_i_ is the fluorescence intensity from Alexa488-MH3-B1/rGel and LTR in each pixel, respectively, and Ch1_avg_ and Ch2_avg_ are the average intensity values of the two chromophores [27]. All calculations were made from nine measured cells from three independent experiments.

### PCI of MH3-B1/rGel *in vivo*

All handling of animals were performed according to standards set by the institutional animal care and use committee, in compliance with the Norwegian Animal Research Authority's guidelines. Cultured subconfluent SKOV-3 cells were injected subcutaneously on the left leg of Hsd:Athymic Nude-*Foxn1^nu^* female mice (5×10^6^ cells/animal). At a tumor size of 75-225 mm^3^, the mice were randomly assigned to 4 treatment groups; no treatment, MH3-B1/rGel, photochemical treatment and PCI of MH3-B1/rGel. The PS TPCS_2a_ was administered intravenously at 5 mg/kg 72 hrs prior to light exposure and 2 mg/kg (100 μl of 0.534 mg/ml stock solution) of MH3-B1/rGel was injected intravenously 24 hrs or 48 hrs before light exposure. Tumors were illuminated by a 652 nm diode laser (CeramOptec GmbH, Bonn, Germany) equipped with a laserfiber (Med*light* SA, Ecublens, Switzerland) at an irradiance of 90 mW cm^−2^ and a total dose of 20 J cm^−2^. Sevoflurane inhalation gas was used as an anesthetic during light exposure and body temperature was maintained by placing the animal on a water-heated plate. The mice were covered by aluminum foil during light exposure, exposing only the tumor area and a ~2 mm margin to light. Tumor volume, measured by a caliper, and weight were monitored 2-3 times a week. Tumor volume was calculated by the equation; 0.5 x *L* x *W^2^* (L = length and W = width of tumor). Animals were euthanized by cervical dislocation when tumor volume exceeded 1000 mm^3^.

### Assessment of liver and kidney toxicity

The animals were divided in 2 groups: no-treatment and PCI of MH3-B1/rGel with 4 animals in each group. The animals were anesthetized and subjected to cardiac puncture 24 hrs after light exposure when 1 ml whole blood was transferred with a 1 ml syringe (27 G Kidneys) to a 4 ml LH Lithium Heparin Separator (Greiner Bio One, Austria). The tubes were subjected to a 15 min centrifugation at 2500 G and serum aspartate aminotransferase (AST), alanine aminotransferase (ALT) and creatinine were assayed on a Cobas6000 (Roche Diagnostics, Mannheim, Germany) within 1.5 hrs after blood sampling. and liver were removed immediately after cardiac puncture and fixed 3 days in formalin prior to H&E staining as previously described [21]. One of the PCI of MH3-B1/rGel treated animals was excluded from the study due to difficulties with the cardiac puncture.

### Statistical analysis

Sigmaplot™ version 12.5 (Systat Software Inc., San Jose, CA, USA) and IBM SPSS (IBM Corporation, Armonk, New York, US) were used for statistical analysis. A 2-sided Student's t-test was used to measure statistical differences and a value of *p* < 0.05 was considered statistically significant.

## SUPPLEMENTARY MATERIAL AND FIGURE



## Abbreviations

ALT: Alanine aminotransferase
AST: Aspartate aminotransferase
HER2: Human epidermal growth factor receptor 2
LTR: Lysotracker
mAb: Monoclonal antibody
PCI: Photochemical internalization
PE: Pseudomonas exotoxin
PS: Photosensitizer
rGel: recombinant gelonin
RIP: Ribosome-inactivating protein
TI: Targeting index
TKI: Tyrosine kinase inhibitor
TPCS_2a_: Meso-tetraphenylchlorin with two sulfonate groups on adjacent phenyl rings
