# Supplementary material for: Photochemical activation of MH3-B1/rGel: a HER2-targeted treatment approach for ovarian cancer
Source: Oncotarget. 2015 Apr 14;6(14):12436–51. doi: 10.18632/oncotarget.3814 (PMC4494949; doi:10.18632/oncotarget.3814)
Supplement: Supplementary file 1 [file oncotarget-06-12436-s001.pdf]

Photochemical activation of MH3-B1/rGel; a HER2-targeted treatment approach for ovarian cancer

Supplementary Material

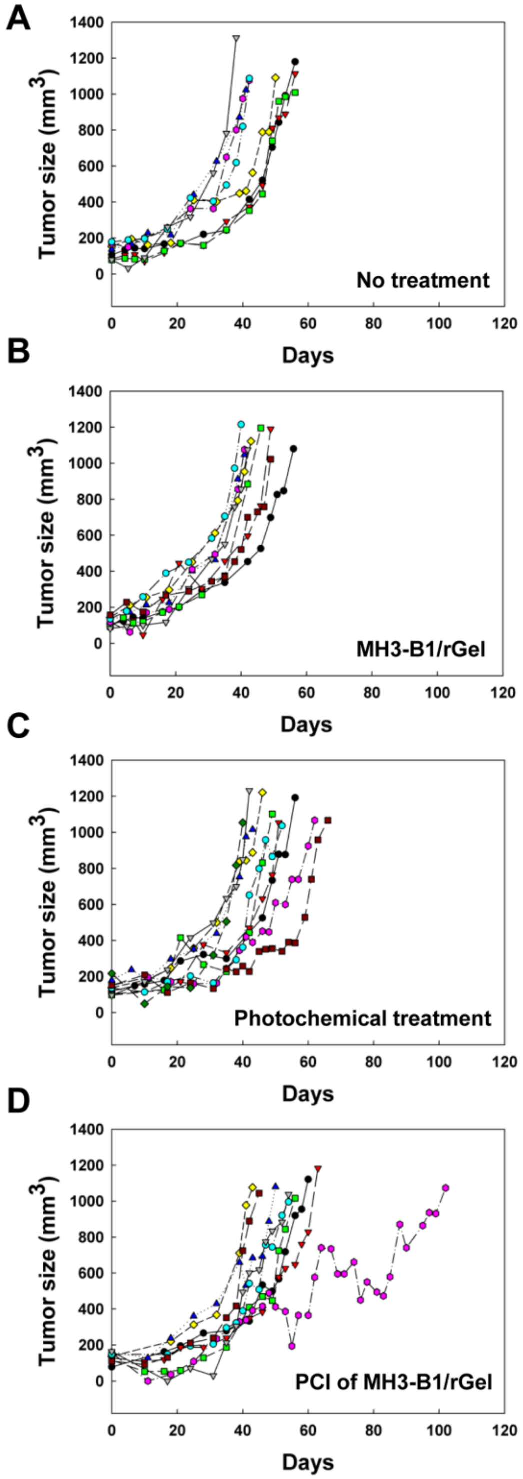

Fig. S1.
Growth curves of SKOV-3 xenografts in the four treatment groups; no treatment (A), MH3-B1/rGel (B), photochemical treatment (C) and PCI of MH3-B1/rGel (D).
